# Editorial: Advances in and Application of Robotic-Assisted Surgery for Colorectal Cancer

**DOI:** 10.3389/fonc.2021.753880

**Published:** 2021-09-22

**Authors:** Po-Jung Chen, Jaw-Yuan Wang, Baoqing Jia

**Affiliations:** ^1^Division of Colorectal Surgery, Department of Surgery, Kaohsiung Medical University Hospital, Kaohsiung Medical University, Kaohsiung, Taiwan; ^2^Department of Surgery, Faculty of Medicine, College of Medicine, Kaohsiung Medical University, Kaohsiung, Taiwan; ^3^Graduate Institute of Clinical Medicine, College of Medicine, Kaohsiung Medical University, Kaohsiung, Taiwan; ^4^Colorectal Surgery Department, Ministry of Health and Welfare Pingtung Hospital, Pingtung, Taiwan; ^5^Pingtung Hospital, Ministry of Health and Welfare, Pingtung, Taiwan; ^6^Department of General Surgery, The First Medical Centre, Chinese PLA General Hospital, Beijing, China

**Keywords:** colorectal cancer, robotic surgery, selective ligation of IMA, anastomotic leakage, anal dysfunction management

Robotic-assisted surgery has evolved quickly and changed how surgery has traditionally been conducted. Compared with laparoscopic colorectal surgery, robotic-assisted surgery offers many advantages, such as instruments that can rotate and bend in all directions, three-dimensional high-definition vision, and surgeon-controlled multi-arms. Therefore, robotic-assisted surgery is advantageous for cases of obesity or a narrow pelvis.

Learning to use robotic technology involves a steep learning curve and longer operating time. The number of cases required to achieve expertise in robotic surgery has been reported in several studies. In the review of Yao et al., the learning curve for robotic NOSES was achieved after the 42nd and 15th cases for Yao and Li, respectively. However, patient selection seems to be one of the most crucial factors influencing the perioperative outcome of colorectal robotic surgery. Müller et al. noted a learning curve of approximately 40 cases is reasonable. Additionally, they noted that not only case load but also case complexity should be considered key factors for the safe implementation of robotic surgery into clinical practice.

High dissection and the selective ligation technique (Yin et al.) seem to result in a lower anastomosis leakage rate and yield favorable clinical and oncologic outcomes in rectal or sigmoid colon cancer treatment. The preservation of the left colic artery and ligation of sigmoid or superior rectal artery is accomplished easily under robotic assistance. Whether the inferior mesenteric vein is ligated depends on the tension during colonic anastomosis. It is an uncommon practice in East Asian countries to ligate the IMV when performing robotic-assisted low anterior resection.

When performing robotic intersphincteric resection with coloanal anastomosis, one of the most important consideration is maintaining patient quality of life. Complications such as anal dysfunction and sphincter muscle injury could occur in patients. Xiaosong et al. proposed a comprehensive management procedure. This procedure includes low-frequency anal electrical biofeedback treatment and daily endoanal suppository usage, which may accelerate sphincteric function recovery and anal sensitivity after robotic total ISR, thereby helping to avoid permanent stoma and greatly improving patients’ quality of life.

Anastomotic leakage is another serious problem in robotic-assisted colorectal surgery. Chen et al. observed that being male, having a lower tumor location (5 cm from the anus), and having a prolonged operation time were independent risk factors for anastomotic leakage in middle and lower rectal cancer with robotic surgery. However, whether neoadjuvant chemoradiotherapy causes anastomotic leakage requires further study before confirmation.

A new type of robotic platform, Micro Hand S (Zeng et al.), which shares some features with da Vinci Surgical Systems, was developed in China. It has only two arms, and therefore, requires an assistant to hold the scope. Compared with da Vinci Surgical Systems, it costs considerably less and has similar oncologic outcomes when used to perform robotic-assisted total mesorectal excision.

This editorial describes the status of information relevant to robotic-assisted colorectal surgery in a variety of clinical categories to share surgical skills, experience, and methods; to improve surgical efficiency; and to promote robotic colorectal cancer surgery. The contributions of this editorial have the potential to improve outcomes in robotic-assisted colorectal surgery.

## Author Contributions

PJ-C contributed to the writing, and J-YW and BJ reviewed the final version of this editorial. All authors contributed to the article and approved the submitted version.

## Conflict of Interest

The authors declare that the research was conducted in the absence of any commercial or financial relationships that could be construed as a potential conflicts of interest.

## Publisher’s Note

All claims expressed in this article are solely those of the authors and do not necessarily represent those of their affiliated organizations, or those of the publisher, the editors and the reviewers. Any product that may be evaluated in this article, or claim that may be made by its manufacturer, is not guaranteed or endorsed by the publisher.

